# A genome-wide screening uncovers the role of CCAR2 as an antagonist of DNA end resection

**DOI:** 10.1038/ncomms12364

**Published:** 2016-08-09

**Authors:** Ana López-Saavedra, Daniel Gómez-Cabello, María Salud Domínguez-Sánchez, Fernando Mejías-Navarro, María Jesús Fernández-Ávila, Christoffel Dinant, María Isabel Martínez-Macías, Jiri Bartek, Pablo Huertas

**Affiliations:** 1 Departamento de Genética, Universidad de Sevilla, 41080 Sevilla, Spain; 2 Department of Regenerative Medicine, Centro Andaluz de Biología Molecular y Medicina Regenerativa, 41092 Sevilla, Spain; 3 Genome Integrity Unit, Danish Cancer Society Research Centre, Strandboulevarden 49, 2100 Copenhagen, Denmark; 4 Department of Medical Biochemistry and Biophysics, Science for Life Laboratory, Karolinska Institute, 171 76 Stockholm, Sweden

## Abstract

There are two major and alternative pathways to repair DNA double-strand breaks: non-homologous end-joining and homologous recombination. Here we identify and characterize novel factors involved in choosing between these pathways; in this study we took advantage of the SeeSaw Reporter, in which the repair of double-strand breaks by homology-independent or -dependent mechanisms is distinguished by the accumulation of green or red fluorescence, respectively. Using a genome-wide human esiRNA (endoribonuclease-prepared siRNA) library, we isolate genes that control the recombination/end-joining ratio. Here we report that two distinct sets of genes are involved in the control of the balance between NHEJ and HR: those that are required to facilitate recombination and those that favour NHEJ. This last category includes CCAR2/DBC1, which we show inhibits recombination by limiting the initiation and the extent of DNA end resection, thereby acting as an antagonist of CtIP.

## Abstract (web summary)

A DNA double strand break can be repaired through either the non-homologous end-joining or the homologous recombination pathways. Here the authors conduct a genome-wide screen and identify a role for CCAR2 in pathway choice by regulating DNA end resection by CtIP.

## Graphical abstract

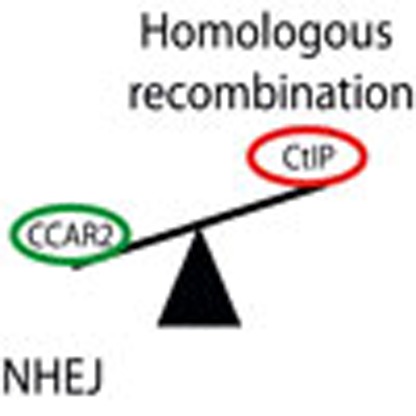

DNA double-strand breaks (DSBs) are the most dangerous form of DNA damage. Unrepaired breaks lead to cell death, while improperly repaired breaks cause an increase in genomic instability and, in humans, diseases such as cancer and premature aging^1^,^2^. There are two major pathways to repair DSBs: non-homologous end-joining (NHEJ) and homologous recombination (HR)^3^,^4^,^5^. NHEJ consists of a ligation of two DNA ends without using homology^5^. In HR, a homologous sequence is used as an information donor in a highly regulated mechanism^3^. Several recombination subpathways have been described, each one with distinct outcomes and consequences^3^. The choice between these two repair mechanisms is highly regulated, and changes in the ratio between them can increase genomic instability^4^. So far, the best-known regulated step of the DSB repair pathway choice is the so-called DNA end resection^4^. Here strands are degraded 5′–3′ at each DNA end, giving rise to ssDNA tails that are immediately coated by the replication protein A (RPA) complex for protection^4^. RPA-coated ssDNA is an obligatory substrate of HR and hampers NHEJ^4^. A major player in the choice between NHEJ and HR is CtIP (CtBP interacting protein), which licenses HR by activating DNA end resection^6^. Multiple signals converge on CtIP to initiate DNA end resection at those breaks that will be repaired by HR^4^,^6^,^7^. In order to find and characterize new factors involved in this crucial choice, we took advantage of the SeeSaw Reporter (SSR), a system designed to assess the balance between NHEJ and HR^8^. Using a genome-wide human esiRNA library, we found that downregulation of 1.35% of the genes shifts the NHEJ:HR ratio towards NHEJ, while depletion of a further 0.71% has the opposite effect. We focused on CCAR2, which we found to cause hyper-recombination when depleted. We show that it acts as an inhibitor of recombination. Specifically, we found that CCAR2 inhibits initiation and limits the extent of DNA end resection through its functional interaction with CtIP. This regulation of DNA end processing modulates the choice between NHEJ and HR.

## Results

### A genome-wide screening for regulators of the NHEJ:HR ratio

**Figure 1:**
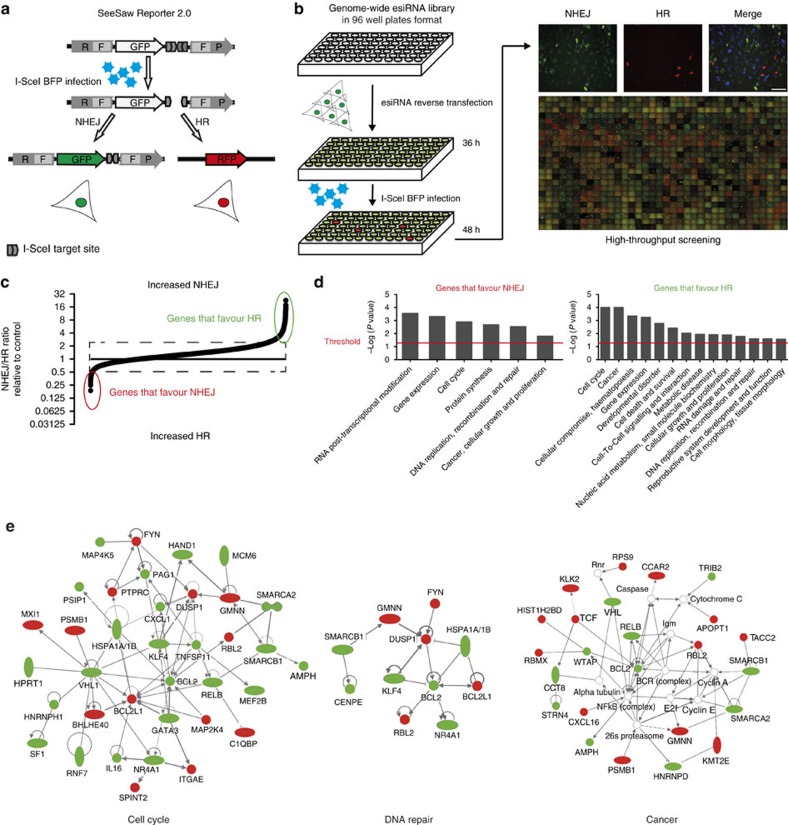
A genome-wide screening for factors that control the balance between NHEJ and HR. (**a**) Schematic representation of the SSR. An I-SceI-induced DSB can be repaired by NHEJ, thus reconstructing an active GFP gene, or by homologous recombination using RFP fragments, thus creating a functional RFP gene. (**b**) Workflow of the screening method. Individual esiRNAs deposited in a 96-well plate format were used to reverse-transfect U2OS cells harbouring a single copy of the SSR system. After 36 h, lentiviral particles bearing I-SceI and the BFP genes were transduced into cells. After 2 days, cells were imaged using a high-throughput microscope for green, red and blue fluorescence. The number of green and red cells was then established for each individual esiRNA. Scale bar, 100 μm (**c**). The ratio between green and red cells was established for each individual esiRNA and normalized to an internal control (esiRNA targeted against luciferase) that was included in each plate. The individual NHEJ:HR ratio was then ordered and plotted. Cells with a normalized ratio close to one occupy the central dashed rectangle. Genes for which depletion reduced the ratio (that is, HR was increased) are marked in the red ellipse and correspond to proteins that naturally favour NHEJ. In contrast, genes that encode pro-recombination proteins (that is, NHEJ increased when they were downregulated) are included inside the green ellipse. (**d**) Functional categories enriched among candidates. The list of candidates that alter the NHEJ:HR ratio was analysed using the IPA Software and categorized into functional groups. Those categories in which genes were statistically significantly over-represented in the set of genes that favour NHEJ (left) or HR (right) are plotted. (**e**) Network of genes involved in *cell cycle, DNA repair, replication and recombination* and *cancer*, which appear in the set of candidates isolated in the screening. Genes that favour HR are labelled in green, and those that facilitate NHEJ, in red.

The SSR2.0 system (Fig. 1a) was designed to calculate the balance between NHEJ and HR as the ratio of green fluorescent protein (GFP)-positive versus red fluorescent protein (RFP)-positive cells of a lone DSB induced by the meganuclease I-SceI (ref. 8). Note that, in this reporter, mainly a specific subtype of HR termed single-strand annealing (SSA) is measured, which is Rad51-independent and does not require strand invasion^3^. SSA is very sensitive to DNA end resection but does not require additional steps; thus, our screening focused on the early steps shared by the various HR subpathways. We measured the ratio of green versus red cells using a high-throughput microscope after individually downregulating human genes using a genome-wide esiRNA library (Fig. 1b). We used 96-well plates and included esiRNA against luciferase in each plate as a control. We discarded the results of any plate in which the green versus red cell ratio of the luciferase control varied more than 10% relative to the average value from all luciferase controls. The ratio of green versus red cells was calculated for each esiRNA and normalized with the value of the internal esiRNA against luciferase. The experiment was repeated independently three times (Supplementary Data 1). Genes were ranked accordingly to average GFP:RFP cell ratio normalized with luciferase and represented graphically (Fig. 1c). We observed three categories of genes with respect to the shape of the curve. Downregulation of the majority of the genes showed a NHEJ:HR ratio similar to the control (for example, normalized GFP:RFP ratio close to 1; dashed black rectangle, Fig. 1c). Depletion of 0.71% of the genes skewed the balance towards an increase in HR (for example, normalized GFP:RFP ratio below 0.5; red ellipse, Fig. 1c). As downregulation of those genes increased HR, we categorize them as genes that naturally favour NHEJ. An additional 1.35% of the genes favoured HR, that is, NHEJ increased when downregulated (for example, normalized GFP:RFP ratio above 3; green ellipse, Fig. 1c). The thresholds of 0.5 and 3 were established with respect to the inflection points of the curve. Data analyses revealed false-positive signals for some genes because of a single experiment with extreme values. To eliminate those, we established the following criteria (Supplementary Data 2): genes for which depletion caused an average normalized GFP:RFP ratio below 0.5, with an individual GFP:RFP normalized ratio below 0.75 for all three replicas, were included in the category of genes that favour NHEJ. In contrast, genes for which depletion caused an average normalized GFP:RFP ratio above 3, with an individual GFP:RFP normalized ratio above 2 for all three replicas, were included in the category of genes that favour HR.

Cell cycle is a major regulator of DSB repair pathway choice, as DNA end resection is limited to the S and G2 phases. Thus, any genes whose downregulation has an impact on cell cycle distribution might indirectly affect the NHEJ:HR ratio. To discard those cases, we used FUCCI-U2OS cells, in which the cell cycle distribution can be visualized under the microscope because of the accumulation of cell cycle-controlled protein fragments fused to fluorescent markers^9^. We used 358 candidate esiRNAs (Supplementary Data 2) to transfect FUCCI-U2OS cells. The percentage of G1 cells was determined by the expression of orange-labelled Cdt1. Cell cycle distribution was normalized to control cells transfected with esiRNA against luciferase to obtain a cell cycle correction factor. We then adjusted the NHEJ:HR ratio according to cell cycle distribution (Supplementary Data 3). Cell cycle correction had little effect on the NHEJ:HR ratio in the majority of cases, and only 18 genes were discarded. In other cases, the corrected ratio was even more robustly shifted. Thus, after considering cell cycle, we ended up with a list of 117 genes that favour NHEJ and 223 genes that favour HR (Supplementary Data 3).

### Network analysis

We next analysed these candidate genes *in silico* by evaluating whether they were over-represented in certain functional categories defined automatically using the IPA software (Ingenuity Systems). Both the genes that favour NHEJ and those that favour HR were enriched in the functional categories *Cell Cycle* and *DNA Replication, Recombination and Repair* (Fig. 1d). Moreover, and in agreement with previous results, RNA metabolism is related to the balance between HR and NHEJ^10^ (Fig. 1d). We also found that Cancer-related genes were over-represented in both sets of genes (Fig. 1d), validating the relevance of balanced DSB repair for avoiding tumorigenesis. The Cell Cycle, DNA Repair and Cancer networks are shown (Fig. 1e).

### CCAR2 is a bona fide regulator of DSB repair pathway choice

The gene for CCAR2 (Cell Cycle and Apoptosis Regulator 2) was ranked first among the candidates that favour NHEJ (Supplementary Data 3). CCAR2 is also known as DBC1 (Deleted in Breast Cancer 1) and KIAA1967. We thereafter focused in the role of CCAR2 in DSB repair pathway choice.

**Figure 2:**
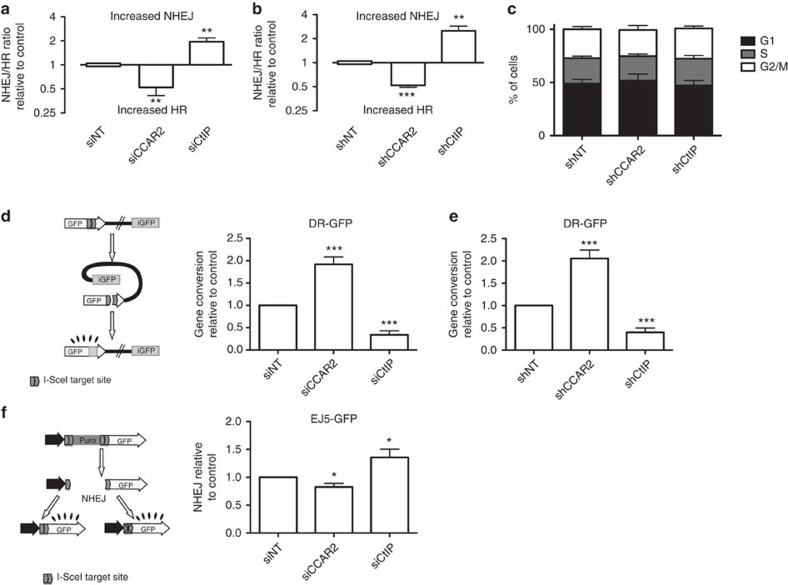
CCAR2 depletion leads to hyper-recombination. (**a**) Cells harbouring a single copy of SSR were transfected with the indicated siRNAs. A non-target siRNA (siNT) and an siRNA against CtIP were used as negative and positive controls, respectively. The resultant NHEJ:HR ratio was normalized to control cells and plotted. The average and s.d. of four independent experiments performed in triplicate are shown. Statistical significance is marked with one to three asterisks, as described in the Methods section. (**b**) Same as **a**, but with cells infected with lentiviral particles harbouring the indicated shRNAs. shNT (a non-target shRNA) and shRNA against CtIP were used as negative and positive controls, respectively. (**c**) Cell cycle distribution of cells after downregulation of the indicated genes with shRNA. (**d**) A schematic representation of the HR reporter DR-GFP is shown on the left side. An intramolecular gene conversion is shown, although it is possible that such reporter engages in an intermolecular recombination event with the sister chromatid, with the same results. Cells bearing such reporters were transformed with the indicated siRNAs. Further details are as in **a**. (**e**) Same as **d**, except that cells were transduced with viral particles containing the indicated shRNAs. (**f**) A schematic representation of the NHEJ reporter EJ5-GFP is shown on the left side. Cells bearing such a reporter were transformed with the indicated siRNAs. Further details are as in **a**.

We initially validated the unbalance between HR and NHEJ using the SSR system in cells depleted of CCAR2 by using different short interfering RNA (siRNA) and short hairpin RNA (shRNAs; for CCAR2 depletion, see Supplementary Fig. 1a,b). CCAR2 downregulation increased HR at the expense of NHEJ in all cases (Fig. 2a,b). We used the DNA resection gene CtIP as a positive control. The shift towards HR with CCAR2 downregulation was not because of a change in cell cycle distribution (Fig. 2c). As the SSR system measures specifically the SSA type of HR^8^, which does not require strand invasion^3^, we tested whether the hyper-recombination observed upon CCAR2 downregulation was specific for the SSA HR subpathway or whether it is a general feature. For this, we tested the effect of its depletion on the reporter DR-GFP, which render cells GFP-positive if gene conversion has occurred after an I-SceI-induced DSB but not if the break was repaired by SSA^11^ (Fig. 2d,e). CCAR2 also inhibited gene conversion. We confirmed that the main contribution of CCAR2 in DSB repair balance is to control HR, as only a mild decrease in NHEJ was observed on the NHEJ reporter EJ5-GFP^12^ (Fig. 2f). Thus, CCAR2 is a bona fide general inhibitor of HR in human cells.

CCAR2 has been shown to control the response to cellular stress by reducing SIRT1 activity and p53 acetylation^13^,^14^. Thus, one possibility was that CCAR2 depletion caused an upregulation of SIRT1 activity, leading to an increase in HR. In that scenario, decrease in SIRT1 should have the opposite effect, that is, reduce HR. We discarded this hypothesis, as SIRT1 depletion actually increases recombination (Supplementary Fig. 2). Hence, we conclude that CCAR2 inhibits HR in a SIRT1-independent manner.

### CCAR2 recruitment to sites of DNA damage

**Figure 3:**
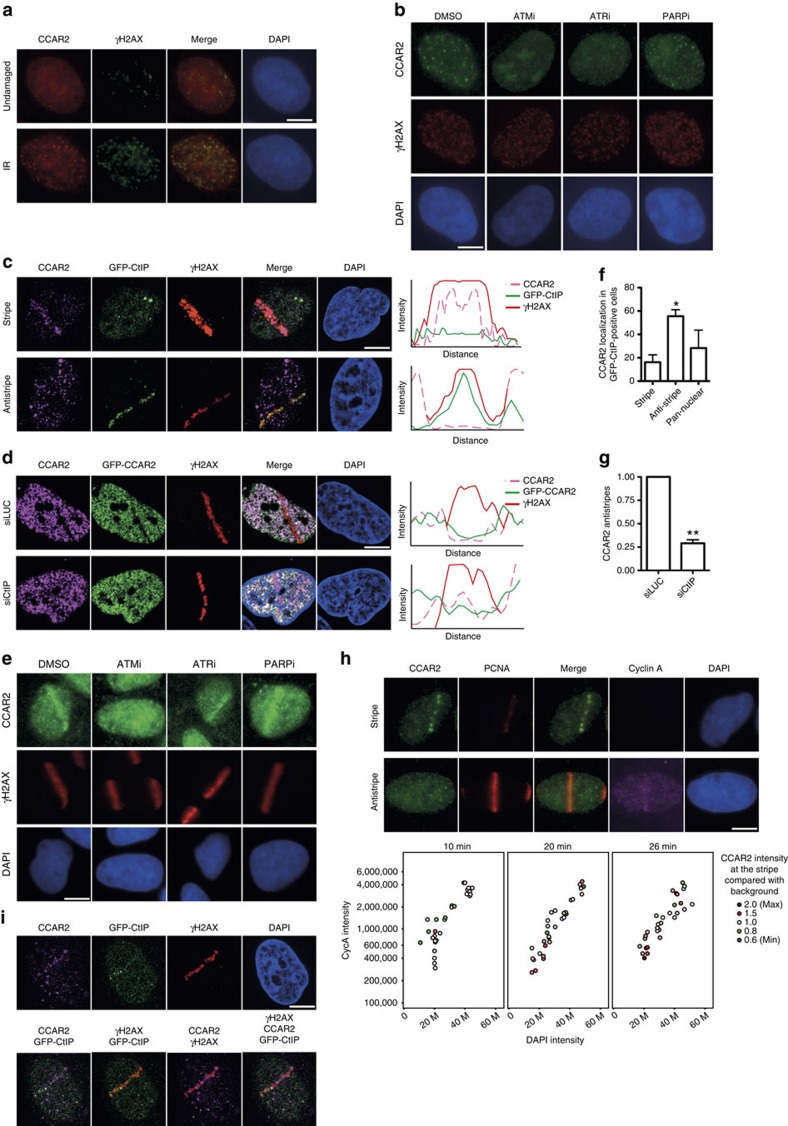
Opposite recruitment of CCAR2 and CtIP to damaged chromatin. (**a**) CCAR2 (red) and the phosphorylated form of H2AX (γH2AX; green) were immunodetected in cells untreated or exposed to 10 Gy of IR. (**b**) Cells pretreated with inhibitors against ATM (ATMi), ATR (ATRi), PARP (PARPi) or DMSO as a control were irradiated and used for immunofluorescence against CCAR2 and γH2AX. (**c**) Cells expressing a GFP–CtIP fusion were microirradiated. CCAR2 recruitment or exclusion from damaged chromatin was determined with an anti-CCAR2 antibody (magenta). Damaged DNA was visualized using an antibody against γH2AX (red). CtIP was observed as accumulation of GFP signal. The intensities of the signals of the CCAR2 antibody, GFP-CtIP and γH2AX were determined by an orthogonal line that crossed the damaged chromatin and plotted. (**d**) Same as in **c**, but in cells bearing a GFP-CCAR2 construct and transfected with siRNA against CtIP or luciferase, as indicated. (**e**) Same as in **c**, but cells were pretreated with the mentioned inhibitors. (**f**) Percentage of cells with GFP–CtIP recruitment that showed recruitment, exclusion or pan-nuclear staining of CCAR2. The average and s.d.'s of three independent experiments are shown. Statistical significance is marked with one to three asterisks, as described in the Methods section. (**g**) Number of CCAR2 antistripes upon depletion of CtIP. Data were normalized with an siLUC. The average and s.d. of three independent experiments are shown. (**h**) Cells were harvested at the indicated times after laser microirradiation and stained for PCNA (red), CCAR2 (green), Cyclin A (magenta) or DNA (DAPI; blue). The nuclear intensity of the DAPI and Cyclin A of individual cells (represented as coloured circles) was measured and plotted to follow cell cycle progression. The intensity of CCAR2 at damaged chromatin (automatically detected as PCNA stripes) was compared with background levels and plotted in red (intensity at the laser tracks over background; that is, CCAR2 stripes) or green (intensity at the laser tracks below background; that is, CCAR2 antistripes) according to the legend. Representative images are shown on top. (**i**) A representative cell with both CtIP (green) and CCAR2 (magenta) stripes is shown. γH2AX is shown in red. Images merging two or three colours are shown. In all panels, scale bars, 7.5 μm.

Proteins involved in DSB repair commonly act locally at the vicinity of damaged DNA. Such activity can be visualized by the local accumulation of the protein to damaged chromatin. Indeed, CCAR2 changed its localization when cells were challenged with ionizing radiation, after which it accumulated at DSBs labelled by the presence of H2AX phosphorylated at Serine 139 (γH2AX; Fig. 3a). Recruitment of DNA repair proteins to DNA damage usually depends on the activation of the DNA damage response. To understand in more detail CCAR2 retention at broken DNA, we used inhibitors targeting the two major kinases that trigger the damage response, ATM and ATR, as well as the poly(ADP-ribose) polymerase (PARP). ATM inhibition completely abolished CCAR2 recruitment upon exposure to ionizing radiation, whereas ATR inhibitor only partially reduced CCAR2 accumulation (Fig. 3b). Note that this effect was evident even for cells that retained some γH2AX despite the ATM inhibition. In contrast, cells treated with PARP inhibitors behaved as control cells following radiation.

Only a subset of γH2AX foci colocalized with CCAR2 foci (Fig. 3a). To monitor CCAR2 recruitment at a higher resolution, we used a laser microirradiation (Fig. 3c). Using an antibody against CCAR2, we observed that indeed it accumulates at sites of DNA damage in 7% of cells (CCAR2 stripe; Fig. 3c). In contrast, we observed that damaged chromatin, labelled with γH2AX, was devoid of CCAR2 in 37% of the cells (CCAR2 antistripe; Fig. 3c). In the remaining cells (56%), we did not observe any specific recruitment pattern; however, we cannot determine for those cells whether CCAR2 was not mobilized upon DNA damage, whether the difference between its signal and background was too low to be distinguished or whether the combination of recruitment and exclusion on the same laser lines prevent its visualization. We interpreted such results as showing that CCAR2 is recruited to DNA damage in a subset of breaks but is actively excluded in another subset. Other proteins that form both stripes and so-called antistripes upon laser microirradiation have been already described^10^,^15^,^16^. We confirmed the existence of CCAR2 antistripes by repeating the experiment in cells stably expressing GFP-CCAR2 (Fig. 3d). Treatment of cells with inhibitors against ATM, ATR or PARP before laser microirradiation rendered a similar picture as for CCAR2 foci formation (Fig. 3e). Thus, CCAR2 laser stripes, but not antistripes, are completely dependent on ATM, partially dependent on ATR and independent of PARP activity (Fig. 3e).

An interesting hypothesis is that cells that use NHEJ for repair recruit CCAR2 to inhibit HR, while cells that use HR for repair exclude CCAR2 from damaged chromatin to allow recombination to take place. Indeed, CCAR2 immunostaining appeared to be mutually exclusive with accumulation of the pro-resection protein CtIP in cells harbouring a GFP-CtIP construct. Further, more than 50% of cells that accumulated CtIP showed clear CCAR2 exclusion from damaged chromatin (Fig. 3c,f). In contrast, the majority of the cells that showed CCAR2 stripes did not show GFP-CtIP accumulation (Fig. 3c). We observed colocalization in less than 20% of the cells (Fig. 3f). Moreover, the formation of CCAR2 antistripes was reduced by fourfold after CtIP depletion in GFP-CCAR2 cells (Fig. 3d,g). We conclude that CtIP was recruited and retained to DNA damage independently of CCAR2, but that CCAR2 antistripes depended on either CtIP accumulation or CtIP-mediated resection.

DNA resection is constrained to the S and G2 cell cycle phases because of, among other things, the phosphorylation of CtIP by cyclin-dependent kinases (CDKs)^4^,^7^,^17^. In order to investigate whether cell cycle position affected CCAR2 retention at, or exclusion from, damaged chromatin, we performed laser microirradiation in cells stained with proliferating cell nuclear antigen (PCNA), to visualize the DNA damage and cyclin A, to follow cell cycle progression. Cyclin A accumulates in S and G2 phases. To discriminate between them, we followed the intensity of the staining with cyclin A, which steadily increases during the cell cycle, and 4′,6-diamidino-2-phenylindole (DAPI), which also increases as a consequence of DNA replication. Thus, G1 cells (low cyclin A and low DAPI signal) evolved into G2 (high cyclin and DAPI signal) going through the S phase (intermediate cyclin and DAPI signals; Fig. 3h). At early time points, we observed higher levels of CCAR2 at damaged chromatin as compared with background (stripes) mainly in G1 and G2 phases but also in the S phase. In contrast, the average intensity at microirradiated lesions was below the background levels (antistripe) mostly in the S phase (Fig. 3h). With time, CCAR2 recruitment became more obvious in G1 and G2, and some CCAR2 exclusion was observed in G2 but never in G1 (Fig. 3h). This suggested that CCAR2 exclusion from DSBs was mainly restricted to replicating cells, with only cells in G1 or G2 able to recruit and retain CCAR2.

CtIP does not form laser stripes in G1 cells^6^. Thus, we hypothesized that cells with both CtIP and CCAR2 stripes would reflect only a proportion of G2 cells. Laser microirradiation causes hundreds of breaks in a given cell. For cells containing laser lines for both CtIP and CCAR2, we wondered whether both were recruited to the same locations. Indeed, when we looked closely at cells with recruitment of both proteins, we observed that the two signals tended not to colocalize (Fig. 3i); this probably reflects the different mechanisms and/or kinetics of repair for DSBs created on the same laser track.

### Resection is inhibited by CCAR2

**Figure 4:**
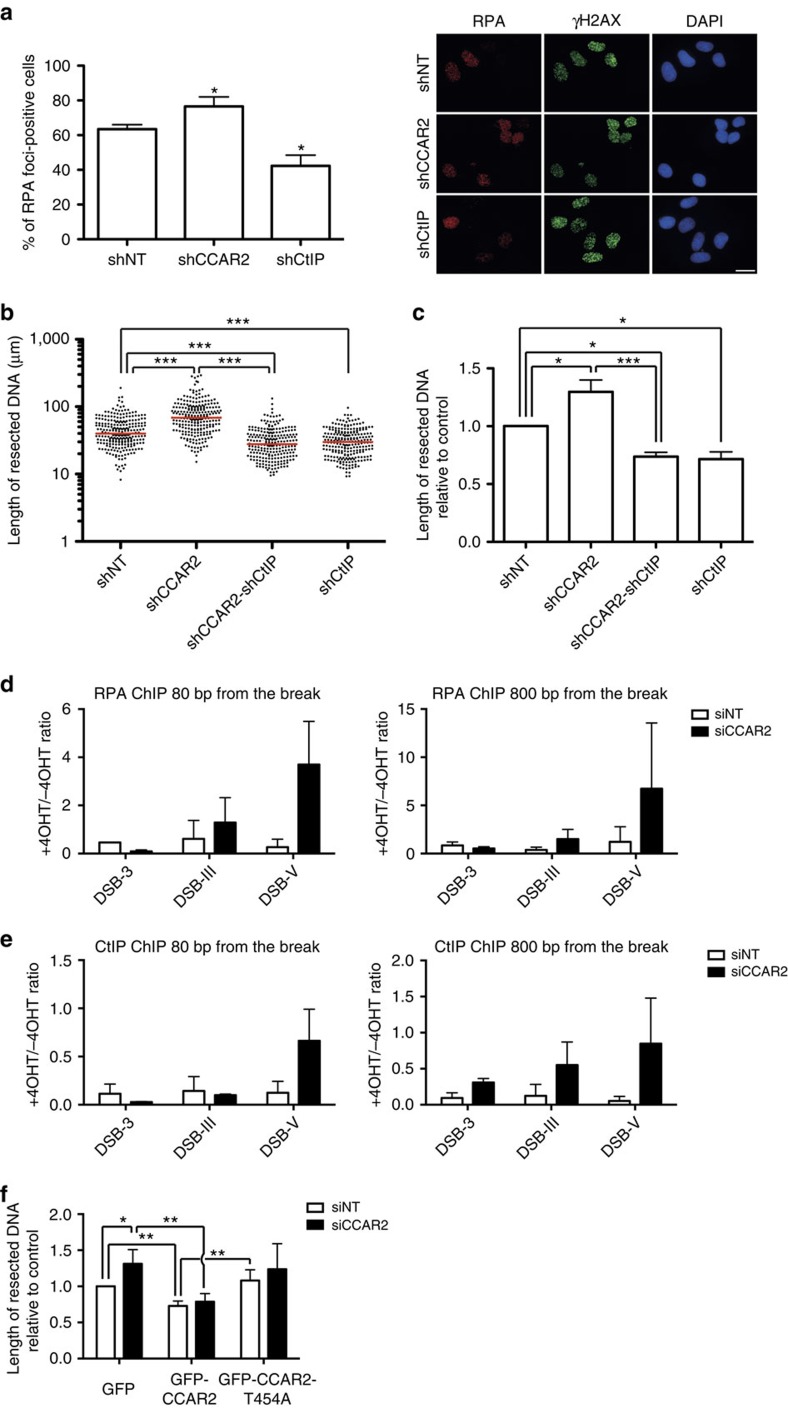
CCAR2 inhibits CtIP-mediated resection. (**a**) Cells transduced with shRNAs against the indicated genes were irradiated (10 Gy). One hour after irradiation, cells were fixed and immunostained as indicated in the Methods section. The number of cells that show RPA foci was scored and represented as a percentage of the total. The graph represents the average and s.d.'s of three independent experiments. Representative images are shown at the right. Scale bar, 20 μm (**b**). The length of resected DNA was calculated using the SMART technique at individual DNA molecules. A Mann–Whitney test was performed to analyse the differences in dispersion. A representative experiment is shown. The median is shown in red. (**c**) The median of the resected DNA lengths was normalized to controls in cells depleted of the indicated proteins. The plot represents the average and s.d. of the normalized medians of four independent experiments. (**d**) DiVA cells were treated with 4-OHT to induce translocation of the nuclease AsiSI to the nucleus or were mock-treated, as described in the Methods section. Chromatin bound to RPA was immunoprecipitated and the occupancy of RPA was detected by qRT–PCR at 80 bp (left) or 800 bp (right) of three DSBs. DSB-3 represents a chromosome break that is exclusively repaired by NHEJ, whereas both NHEJ and HR can repair DSB-III and DSB-V. The same approach was performed in cells depleted for CCAR2 (black bars) or control cells (white bars). (**e**) Same as **d**, but using an antibody against CtIP for ChIP. (**f**) SMART assay with cells expressing the indicated plasmids and transfected with siRNA against CCAR2 (black bars) or a control sequence (siNT, white bars). Further details are as in **c**.

CtIP-mediated resection is a well-known molecular switch that controls the balance between NHEJ and HR^6^,^7^. As our results suggested that CCAR2 might act as an antagonist of CtIP, we next tested whether CCAR2 regulates DNA end resection. Downregulation of CCAR2 slightly increased the number of cells that were positive for RPA foci upon exposure to ionizing radiation (Fig. 4a). There are two non-exclusive ways to affect DNA end resection: increasing the number of cells that initiate resection and increasing the amount of DNA resected at each specific break. RPA foci formation is a good measurement of the former, but it is not sensitive enough to estimate the latter. Thus, as most S and G2 cells initiate resection, and hence show RPA foci, it is hard to observe an increase in the number of cells positive for RPA. To better resolve the increased resection upon CCAR2 depletion, and to test whether CCAR2 also affected the length of resection, we used a high-resolution technique to analyse DNA resection in single molecules *in vivo* (SMART; see Methods section Single-molecule analysis of resection tracks for details)^18^. In short, it is a modified DNA-combing protocol in which ssDNA length is measured on stretched DNA fibres^18^. Downregulation of CCAR2 not only affected the number of cells that resect DNA, but also allowed resection to continue deeper into the chromosomes (Fig. 4b,c). In addition, such hyper-resection was completely dependent on CtIP (Fig. 4b,c), genetically placing them in the same pathway. Both SMART and RPA foci were observed upon exposure to ionizing radiation. To extend our results, and to link them with the hyper-recombination phenotype observed using nuclease-induced DSBs (Fig. 2), we measured the accumulation of RPA at AsiSI-induced breaks by chromatin immunoprecipitation (ChIP; Fig. 4d). Some breaks induced by this enzyme are repaired only by NHEJ, while others are repaired by both NHEJ and HR^19^. Upon CCAR2 downregulation, we saw an increased accumulation of RPA at two different AsiSI-induced breaks that are normally repaired by NHEJ and HR (Fig. 4d; DSB-III and DSB-V) but no effect when we analysed a break that is exclusively repaired by NHEJ (Fig. 4d; DSB-3). The same was observed using different distances of the cleavage site (Fig. 4d). Thus, it seems that CCAR2 limits resection at breaks that are normally resected. To better understand how this occurs, we tested CtIP recruitment at those same sites after forming DSBs by AsiSI. We observed an increase in CtIP recruitment at all the breaks, especially at distances further away from the actual cleavage site (Fig. 4e).

An interesting idea is that CCAR2 might limit resection of those breaks to which it is actively recruited in an ATM-dependent manner. As ATM itself heavily affects DNA end resection, we could not simply inhibit ATM activity. However, it has been shown previously that ATM phosphorylates CCAR2 at threonine 454 (ref. 20). Thus, we measured resection length at cells depleted of endogenous CCAR2 and complemented with either wild-type or the T454A mutant. Expression of the wild-type version of the protein completely suppressed the hyper-resection observed in cells depleted for CCAR2 (Fig. 4f). Indeed, as exogenous CCAR2 expression led to a overexpression of the protein (Supplementary Fig. 1c), the length of resected DNA in cells harbouring the wild-type gene was shorter than in control cells, reinforcing the idea that CCAR2 is antagonistic to DNA end resection (Fig. 4f). This was not observed when the non-phosphorylatable version of the protein was introduced in the cells, indicating that ATM phosphorylation is truly essential for the inhibitory role of CCAR2 on end processing (Fig. 4f). Indeed, although this mutant partially rescued the resection defect, it was not statistically significantly different from the GFP control (Fig. 4f). Thus, CCAR2 limits the extent of DNA that is resected in an ATM-dependent manner.

### CCAR2 antagonizes CtIP by their physical interaction

**Figure 5:**
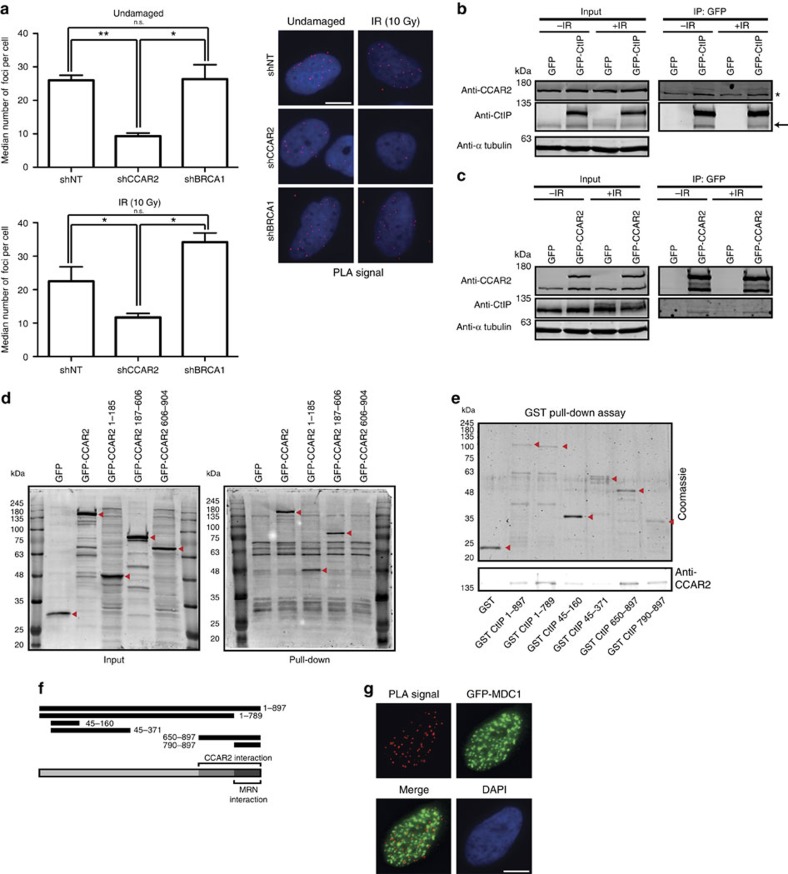
CCAR2 and CtIP interacts physically in a constitutive manner. (**a**) Cells depleted of the indicated genes were analysed with a PLA using CtIP and CCAR2 antibodies. The average and s.d. of the medians of three independent experiments are plotted on the left. A representative image of each condition is shown on the right. The top graph was obtained with cells unchallenged by exogenous damage. The plots on the bottom side were calculated in cells 1 h after irradiation (10 Gy). Scale bar, 7.5 μm. (**b**) Protein samples from cells stably transfected with either GFP or GFP–CtIP in undamaged conditions or 1 h after ionizing radiation (10 Gy) were used for immunoprecipitation with anti-GFP resin. Immunoprecipitates were resolved in SDS–PAGE and blotted for the indicated antibodies. The asterisk marks an unspecific band that binds to the resin, and the arrow marks the endogenous CtIP protein. (**c**) Same as **b**, but with cells expressing GFP or GFP-CCAR2. (**d**) GST–CtIP was used as bait for pull-down experiments from whole-cell extracts using cells expressing GFP, GFP-CCAR2 full-length or three deletion mutants of CCAR2, as indicated. Western blots with an anti-GFP antibody using inputs (left) and pull-downs (right) are shown. The red arrows label the position of GFP fusions. (**e**) Bacterial-purified His_6_-CCAR2 was pulled down with bacterial-purified GST–CtIP full-length and deletion constructs. Purified GST was used as a control. The red arrows represent the purified CtIP version. A western blot against CCAR2 is shown at the bottom. (**f**) A schematic representation of all the deletion constructs used in **e**. Full-length CtIP and the interaction regions with CCAR2 and the MRN complex are represented at the bottom. (**g**) PLA foci using CtIP and CCAR2 antibodies in cells expressing GFP-MDC1 that were collected 1 h after irradiation (10 Gy). Scale bar, 7.5 μm.

To elucidate how CCAR2 exerts its function, we assessed whether CCAR2 and CtIP physically interact. We observed such an interaction using a proximity-ligation assay (PLA; Fig. 5a; Supplementary Fig. 3a; controls for the specificity of the technique are shown in Supplementary Fig. 3b). We wondered whether the interaction is direct or mediated by BRCA1, as both CCAR2 and CtIP have been shown to interact with BRCA1 (refs 21, 22). Depleting BRCA1 did not change the interaction between CtIP and CCAR2 (Fig. 5a). In fact, the CCAR2–CtIP interaction seemed constitutive and did not depend on the presence of DNA damage (Fig. 5a). To confirm the interaction, we purified CtIP from U2OS cells using GFP- and FLAG-tagged version of the protein. Using mass spectrometry, we identified CCAR2 as an interactor of CtIP (see Supplementary Table 1). We observed such an interaction in cells arrested in G1, S and G2. Moreover, using total cell extracts, we were able to co-immunoprecipitate endogenous CCAR2 with GFP-CtIP, both in the presence and absence of DSBs (Fig. 5b and Supplementary Fig. 4a). Note that endogenous CtIP was also specifically immunoprecipitated (Fig. 5b, arrow), in agreement with the ability of CtIP to self-interact^23^. Furthermore, the reciprocal interaction was observed when GFP-CCAR2 was used to immunoprecipitate endogenous CtIP (Fig. 5c and Supplementary Fig. 4b). Remarkably, only the non-phosphorylated form of CtIP (lower band) was co-immunoprecipitated with GFP-CCAR2 from lysates of irradiated cells, in agreement with a damage-independent interaction (Fig. 5c).

In addition, by using purified, bacterial-expressed GST (glutathione *S*-transferase)-CtIP as bait, we were able to pull-down GFP-CCAR2 from whole extracts from human cells (Fig. 5d and Supplementary Fig. 4c). By expressing three truncated versions of GFP-CCAR2, we mapped the interaction region of CCAR2 and CtIP to the first two-thirds of the protein (Fig. 5d and Supplementary Fig. 4c).

We then performed *in vitro* direct binding assays using full-length His_6_-CCAR2 and GST-CtIP purified from bacteria. We detected a direct interaction between the two proteins (Fig. 5e). Using a series of CtIP fragments, we mapped the interaction between CtIP and CCAR2 to the C-terminal part of CtIP (from amino acid 650 to the end of the protein), which covers the region that mediates the interaction of CtIP and the MRN complex (amino acids 790–897)^6^ (Fig. 5e,f). This interaction between CtIP and CCAR2 was at odds with the lack of colocalization of both proteins at laser-induced damage. However, we reasoned that these observations could reflect a general interaction of both proteins in the bulk population in contrast to what occurs with local exclusion at damaged chromatin (Fig. 3c). Hence, we repeated the proximity-ligation assay in cells expressing GFP-MDC1, which is recruited to damaged DNA. We analysed the interaction between CCAR2 and CtIP in the vicinity of the damaged chromatin (that is, in MDC1 foci; Fig. 5g). We clearly observed that the CCAR2–CtIP PLA signal almost never colocalized with DNA damage, in agreement with the two proteins being recruited to laser microirradiation mainly in a mutually exclusive manner.

## Discussion

Using a unique reporter, we identified 340 genes that have a role in maintaining the balance between NHEJ and HR in human cells. We used very stringent conditions for the selection of positive candidates. Thus, some proteins with a role in the DSB repair pathway choice may be missing from our final list (Supplementary Data 3). In fact, several proteins that we knew to affect the SSR are not present in this list, but nonetheless shifted the balance in the predicted manner (see Supplementary Data 1 for details). These include pro-recombination activities such as CtIP, BRCA1, MRE11, BLM and EXO1 as well as NHEJ proteins such as LIG4. Moreover, as our genomic approach does not guarantee downregulation of all the genes in the library, we may have missed some important factors because of insufficient depletion.

Interestingly, we spotted not only genes that are required for HR prevalence over NHEJ but also a class of factors with the opposite role. Thus, we describe a sizeable set of genes whose depletion caused a hyper-recombinant phenotype in human cells. Such factors share similar functions to those required for NHEJ^24^, those that decrease the stability of resection proteins, such as PIN1 (ref. 25) or CDH1 (ref. 26) and those that block resection, such as RIF1, 53BP1, REV7 and HELB^27^,^28^,^29^,^30^. In an approach similar to ours but using the recombination assay DR-GFP, a previous study found that several genes are required for gene conversion^10^. We compared both screenings and found little overlap between the two screenings. This might reflect the aforementioned weaknesses of genome-wide siRNA strategies and the stringent conditions applied to the selection of candidates in both screenings; however, it could also be that the distinct screening strategies targeted different steps in the recombination pathway and thus complemented each other. The DR-GFP screen readily responds to Rad51-dependent strand invasion, and indeed the majority of the isolated hits corresponded to proteins that affect Rad51 expression^10^. In contrast, the SSR system is Rad51-independent and readily reacts to changes in DNA resection^8^. Notably, Adamson *et al*.^10^ did not find any genes that showed hyper-recombination phenotypes upon depletion. In contrast, we clearly isolated factors that favour homology-mediated repair when downregulated (Supplementary Data 3). Hyper-recombinant mutants have been abundantly isolated from other organisms^1^ but rarely from higher eukaryotes. Thus, we present the first comprehensive list of genes that cause a hyper-recombinant phenotype when depleted in human cells.

The majority of genes that affected the SSR ratio belong to the functional categories of *DNA repair, replication and recombination, cell cycle* or *mRNA metabolism*, indicating the relevance of such processes in controlling DSB repair. In terms of pathogenesis, we also found a clear correlation between DSB repair balance and cancer.

We singled out CCAR2 to exemplify this category of genes that have a pro-NHEJ and antirecombination role in the cell. CCAR2 interacts with BRCA1 (ref. 21), a well-known tumour suppressor required for HR^31^ and to modulate the speed of DNA end resection^18^. CCAR2 inhibits BRCA1's transcriptional role^21^, affects cell proliferation by controlling SIRT1 and p53 (refs 13, 14, 20, 32) and is involved in RNA metabolism^33^. In most published studies, the role of CCAR2 depends on its role as a SIRT1 inhibitor, a function that requires the phosphorylation of CCAR2 at threonine 454 (refs 13, 14, 20). However, here we describe an SIRT1-independent function in HR. In agreement, CCAR2 contribution to DNA repair has previously been shown to be SIRT1-independent^32^,^34^. Despite the lack of a direct involvement of SIRT1, this new function of CCAR2 is also dependent on ATM-mediated phosphorylation.

The physical relationship between CCAR2 and BRCA1 suggested the possibility that BRCA1 bridges an interaction between CtIP and CCAR2. However, we have excluded this possibility, as this interaction (measured by PLA) was maintained or even increased in the absence of BRCA1. Here we extend the relationship between CCAR2 and DNA repair by showing CCAR2's direct physical and functionally antagonistic relationship with CtIP. CCAR2 acts as a bona fide inhibitor of DNA end resection: it not only regulates which cells resect their DNA but also limits the length of the produced resected DNA. Indeed, depletion of CCAR2 increases the amount of CtIP recruited to AsiSI-induced DSBs, mostly at locations further away of the actual cleavage site. Hence, we postulate that CCAR2 might constrain the spreading of CtIP along the DNA, thereby spatially confining resection. Interestingly, in a proteomic screen CCAR2 was found to interact with all three subunits of the RPA complex^35^; hence, such an interaction might be required for this role-limiting resection. By doing so, it affects all HR pathways, including the most conservative gene conversion. Although it has a mild effect in inhibiting NHEJ, as is expected when breaks are hyper-resected, we conclude that the major role of CCAR2 in DSB repair balance is to antagonize resection spreading. In agreement, CCAR2 depletion does not affect the recruitment of NHEJ proteins such as 53BP1 (ref. 34). Such a role in HR could contribute to CCAR2 sensitivity to DSB-inducing agents^32^,^36^. Our data are in apparent contrast to a previously published report that proposes CCAR2 to be an enhancer of HR, using a recombination reporter in SW480sn3 cells^36^. However, this might reflect differences in the reporters used, and, more specifically, the length of gene conversion tracks required to render positive colonies. This hypothesis is supported by the fact that CCAR2 not only affects the number of DSBs to be resected but also mainly controls the length of DNA that will be resected, hence modulating gene conversion tracks and crossovers^37^.

**Figure 6:**
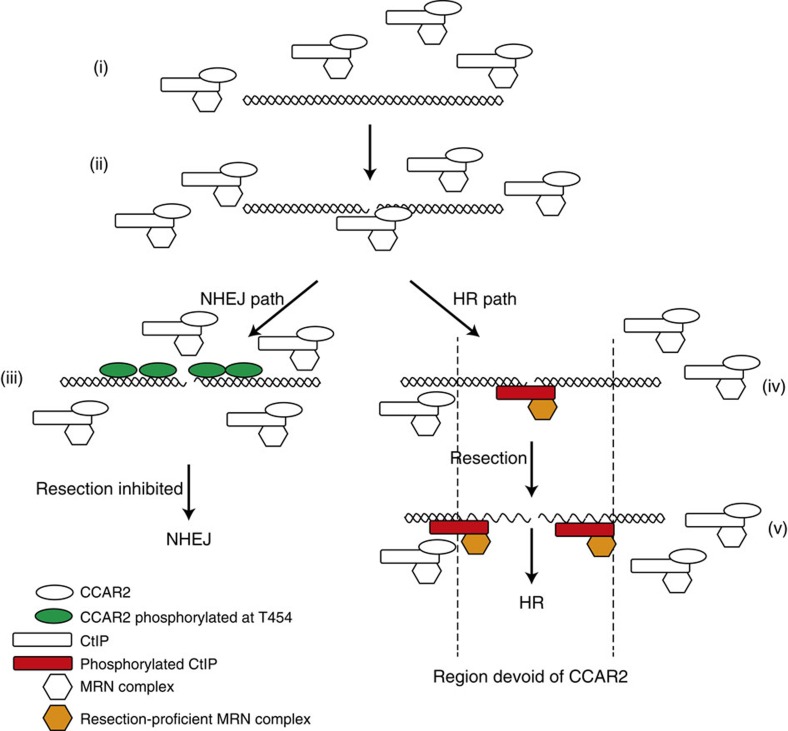
Schematic depiction of the effect of CCAR2 on DNA end resection. CtIP and CCAR2 interact constitutively. Upon the appearance of DSBs, they are both recruited (ii), but soon only one of them is left remaining at the breaks (iii and iv). DSB repaired by NHEJ maintains CCAR2 in an ATM-dependent manner, facilitating repair (iii). In contrast, DSBs that require DNA end resection maintain only CtIP at the break, and, indeed, CCAR2 is completely excluded from the region in a CtIP and/or DNA end resection manner (iv). This allows resection to progress until it covers the CCAR2-free region (v).

Mechanistically, we propose the following model (Fig. 6). CCAR2 interacts constitutively with CtIP in a DNA damage-independent manner in the nucleoplasm (Fig. 6i). Despite the proximity of the CCAR2 and MRN interaction sites in CtIP, there is no competition between the two (Supplementary Fig. 5). Thus, it is more likely that the physical presence of CCAR2 negatively regulates the activity of the CtIP–MRN complex. Upon the appearance of a broken DNA molecule, CCAR2 and CtIP recruitment depends on the cell cycle phase. In G1 cells, only CCAR2 is recruited, as resection will be inactive^4^,^17^. In G2 and (perhaps) S phase, however, they are probably recruited together to chromatin, accounting for the 18% of CtIP laser line-positive cells that also showed CCAR2 accumulation (Fig. 6 ii). The fact that CCAR2 downregulation does not affect CtIP recruitment but only limits its spreading along the DNA, while CtIP depletion reduces CCAR2 exclusion, reinforces the idea that CtIP inhibition by CCAR2 is because of the physical interaction between the two. While the bulk of CtIP and CCAR2 in the nucleoplasm retain their interaction upon DNA damage, the CCAR2–CtIP complex appears to be disrupted locally on damaged chromatin (Fig. 6 iii and iv). Remarkably, CCAR2 interacts only with the non-phosphorylated form of CtIP. In this scenario, either one factor or the other rapidly takes command of the situation. Which one dominates depends on several factors, such as cell cycle distribution or chromatin status. Indeed, CCAR2 affects the kinetics of repair of breaks that occur in heterochromatin but not in euchromatin^34^. Strikingly, CCAR2 has little effect on breaks that are always repaired by NHEJ, but is critical for DSB repair pathway choice for breaks that could be repaired by HR or NHEJ. When a break will be repaired by NHEJ (Fig. 6 iii)—that is, all of the breaks that occur in G1, and many of those in G2–CtIP exits the damaged chromatin, but CCAR2 stays on, constraining DNA end resection and allowing NHEJ to ensue. This retention of CCAR2 and its role as an antagonist of resection requires ATM activity and CCAR2 ATM-mediated phosphorylation at threonine 454 (ref. 20). This parallels the RIF1–53BP1 antiresection pathway, which is also triggered by 53BP1 ATM-mediated phosphorylation^27^,^28^. In contrast, DSBs that will be resected maintain CtIP at sites of DNA damage, and do not accumulate (G2) or even actively exclude (S phase) CCAR2 from these sites (Fig. 6 iv and v), probably thereby allowing the catalytic activity of the MRN complex. Such behaviour of CCAR2 is CtIP-dependent and allows CtIP and DNA end resection to be activated. An interesting hypothesis is that resection will be limited to this chromatin region devoid of CCAR2 (antistripe; Fig. 6v), and will stop as soon as it enters a nuclear region in which CCAR2 is still present. This would minimize the chance of hyper-resection of DSBs, a phenomenon that may be associated with an increase in genomic instability^37^,^38^. The model predicts that the extent of resection in human cells is higher in the S phase than in the G2 phase, a phenomenon that has been observed in budding yeasts^39^. This might explain the increased length of resected DNA tracks upon CCAR2 depletion, as all S- and G2-phase cells would resect their DNA as long as if they were in G2. This will also suggest that HR is probably different between S and G2 cells, as the length of resection will affect the balance between different recombination subpathways, controlling the size of gene conversion tracks and interfering with crossover formations^37^.

We have observed that the CtIP–CCAR2 interaction is independent of BRCA1, suggesting that all pairwise interactions occur independently. This crosstalk between BRCA1, CtIP and CCAR2 might then regulate the fidelity of DNA repair, which could explain why all three of these are related to the appearance of breast cancer yet have opposite effects on HR^40^,^41^,^42^. Thus, CCAR2 is emerging as a critical protein controlling cellular response to stress, including DNA damage, and it elicits a very complex response that involves many parallel reactions.

## Methods

### Cell culture and manipulations

Cells were cultured in high-glucose DMEM supplemented with 10% fetal bovine serum (FBS), 2 mM glutamine, 100 μg ml^−1^ streptomycin and 100 U ml^−1^ penicillin at 37 °C in 5% CO_2_. Plasmids were transfected using FugeneHD Transfection Reagent (Promega) according to the manufacturer's instructions. siRNAs against CCAR2 (5′- GCUUAUAGUUCGAAGGUAC -3′), CtIP (5′- GCUAAAACAGGAACGAAUC -3′), luciferase (5′- CGUACGCGGAAUACUUCGA -3′), SIRT1 (Dharmacon SMARTpool L-003540-00) and a control non-target sequence (a mix of 5′- UGGUUUACAUGUCGACUAA -3′, 5′- UGGUUUACAUGUUGUGUGA -3′, 5′- UGGUUUACAUGUUUUCUGA -3′ and 5′- UGGUUUACAUGUUUUCCUA -3′) were transfected with RNAiMax Lipofectamine Reagent Mix (Life Technologies), according to the manufacturer's instructions. Lentiviruses harbouring shRNA vectors (SIGMA) targeting CtIP (TRCN0000318738), CCAR2 (TRCN0000053723), BRCA1 (TRCN0000009823) or a non-target sequence (SHC016V 01031212MN) were used to infect U2OS cells. Lentiviral particles were obtained as described^8^. Briefly, lentiviral particles were generated by calcium phosphate transfection in A293T cells. After 48 hours, lentiviruses were collected from the media by 100,000*g* centrifugation for 2 hours at 4°C. Cells stably expressing the shRNAs were selected by adding 1 μg ml^−1^ puromycin to the medium after infection. U2OS were a gift from Stephen P. Jackson. FUCCI-U2OS cells were obtained by stably transfecting U2OS cells with pFucci-G1 Orange (AM-V9003) and pFucci-S/G2 Green (AM-V9010) from Amalgaam.

### esiRNA human whole-genome screening

All manipulations were performed using a Hamilton Microlab STAR robot (301–3,811) following a protocol designed by the Genomic Unit of CABIMER. The screening was carried out using the MISSION esiRNA library (SIGMA) targeting 16,538 human genes based on sequence annotation from the ENSEMBL database. The esiRNAs were aliquoted (30 nM) in a 96-well plate format, with each plate harbouring an esiRNA against luciferase as a control. U2OS-SSR2.0 cells were then reverse-transfected at 6,000 cells per well using RNAiMax Lipofectamine Reagent Mix (Life Technologies), according to the manufacturer's instructions. After 6 h, media was replaced to minimize cell death. At 36 h after transfection, I-SceI–BFP (blue fluorescent protein) lentivirus (multiplicity-of-infection of 10) and 6 μg ml^−1^ polybrene diluted in DMEM were added to the cells. Cells were washed the next day with new media; after another 24 h, cells were washed with PBS, fixed with 4% paraformaldehyde and washed twice with PBS. Before staining with Hoechst 33,342, a representative picture was taken to calculate the efficiency of the I-SceI–BFP infection. Plates were then imaged using an ImageXpress Micro (Molecular Devices) at × 10 magnification, and pictures were taken at nine sites (fields) per well. Nuclei, GFP and RFP signals were detected with blue, green and Texas Red filters, respectively. Images were analysed, and the amount of GFP- and RFP-positive cells were quantified automatically with the Image MetaExpress Software (Molecular Devices). The ratio of GFP:RFP cells in each well was normalized using the internal control with esiLUC (Supplementary Data 1).

### Plasmids

GFP-CtIP was published elsewhere^7^. Full-length CCAR2 cDNA was inserted into the pET28a expression vector (Novagen) to add a polyhistidine (His_6_) tag at the N terminus of CCAR2 protein. The cDNA was also cloned into pEGFP-C1 to create the full-length GFP-CCAR2 construct and mutated to avoid silencing with the siRNA. The GFP-CCAR2-T454A mutant was obtained by mutagenesis. CCAR2 deletion mutants were obtained from the full-length construct by cleavage with ApaI and ligation of the obtained fragments in pEGFP-C1 linearized with ApaI (1–185 fragment) or EcoRI-ApaI (187–606 and 608–924 fragments). The full-length pGEX-CtIP construct and CtIP fragment constructs and GFP-MDC1 were a kind gift from Steve Jackson (University of Cambridge, UK).

### Gene conversion:NHEJ and NHEJ:HR balance analysis

U2OS cells bearing a single-copy integration of the reporters DR-GFP (Gene conversion)^11^, EJ5-GFP (NHEJ)^12^ or SSR (NHEJ:HR)^8^ were used to analyse the different DSB repair pathways. In all cases, 4,000 cells were plated in 96-well plates. One day after seeding, cells were transfected with the indicated siRNA or infected with lentiviral particles carrying the indicated shRNA. The medium was changed after 6–8 h. The following day, cells were infected with a lentivirus harbouring I-SceI and labelled with BFP^43^ at an multiplicity-of-infection of 10. After 24 h, cells were washed with fresh medium and maintained during an additional 24 h. Cells were then fixed with 4% paraformaldehyde, stained with Hoechst 33,432 and washed with PBS before visualization with a fluorescent microscope for blue, green and, in the case of SSR, red fluorescence, as described in the previous section. The repair frequency was calculated as the percentage of blue cells expressing GFP for the DR-GFP and EJ5-GFP. For the NHEJ:HR balance, the ratio between green versus red cells in each conditions was calculated as published^8^. To facilitate the comparison between experiments, this ratio was normalized with siRNA or an shRNA control. Conditions that skewed the balance towards increased NHEJ repair resulted in a fold increase above 1. In contrast, a net decrease of this ratio (for example, values below 1) represented an imbalance of SSR towards HR. Data represent a minimum of four sets of triplicate experiments.

### *In silico* analysis

Candidate gene sets were analysed using the IPA Software (Ingenuity Systems, www.ingenuity.com) available through PAB (The Andalusian Platform of Bioinformatics, www.scbi.uma.es) from the University of Malaga. A total of 330 ID genes of 340 candidates were recognized and analysed using the Ingenuity Knowledge database. The core analysis tool was used to visualize functional categories' enrichment and associated networks. Fisher's exact test right-tailed was performed using the IPA Software to calculate *P* values using a cutoff of 0.05.

### Cell cycle analysis using flow cytometer

Cells were harvested, washed with PBS and resuspended in ice-cold PBS. EtOH (70%) was added dropwise while vortexing at low speed, and cells were then fixed at 4 °C for at least 2 h. Cells were washed with PBS and treated with 250 μg ml^−1^ RNase A (SIGMA) and 10 μg ml^−1^ propidium iodide diluted in PBS (Fluka). Cells were incubated at 37 °C for 30 min and analysed using a FACSCalibur (BD).

### Immunofluorescence microscopy

U2OS cells were infected with lentivirus harbouring the indicated shRNA or a control sequence. After 48 h, cells were irradiated (10 Gy) or mock-treated, incubated 1 h for foci formation and collected. For the experiment with inhibitors, ATMi (10 μM), ATRi (5 μM) or PARPi (1 μM) was added to the plates 30 min before irradiation. For RPA foci formation, coverslips were treated for 5 min on ice with pre-extraction buffer (25 mM Tris-HCl, pH 7.5, 50 mM NaCl, 1 mM EDTA, 3 mM MgCl_2_, 300 mM sucrose and 0.2% Triton X-100), and then fixed with 4% paraformaldehyde (w/v) in PBS for 15 min. For CCAR2 foci, cells were fixed with 4% paraformaldehyde (w/v) in PBS for 15 min on ice, washed twice with PBS and permeabilized with 0.2% Triton X-100 (v/v) in PBS for 10 min on ice. Then, coverslips were washed three times with PBS and blocked for at least 1 h with 5% FBS diluted in PBS. Cells were incubated with the adequate primary antibodies (Supplementary Table 2), diluted in 5% FBS in PBS for 2 h at room temperature, washed with PBS and then incubated with secondary antibodies (Supplementary Table 3) diluted in 5% FBS in PBS for 1 h at room temperature. Cells were then washed twice with PBS, and coverslips were mounted with Vectashield mounting medium (Vector Laboratories) containing DAPI and analysed using a LEICA microscope. At least 200 cells were scored per sample. Experiments were repeated independently at least three times.

### Laser microirradiation

For laser microirradiation, cells with the indicated downregulated proteins were grown in medium containing 10 μM bromodeoxyuridine (BrdU, GE Healthcare) for 24 h in glass-bottom dishes or on coverslips. Pretreatment with inhibitors was performed as described in the previous section. A Zeiss PALM Microbeam microscope was used to induce DNA damage with a UV-A (355 nm) laser. Experiments of microirradiated cells were performed in CO_2_-independent, phenol red–free DMEM. Laser output was set to the lowest setting that induced DNA damage, as monitored by phosphorylation of H2AX. Confocal images were obtained with a Leica Microscope TCS 5PS, using the LAS AF Software (Leica). The MetaMorph Software was used to quantify the amount of fluorescence (stripes and antistripes) across a linear pathway transversal to the microirradiated signal, visualized as γH2AX. At least 200 cells positive for γH2AX stripes were counted per sample.

### Proximity-ligation assay

PLAs were performed using the Duolink PLA Kit (Olink Bioscience, Uppsala, Sweden) according to the manufacturer's protocol. Briefly, U2OS cells were infected with lentivirus harbouring the indicated shRNA or a control sequence. After selection in medium with 1 μg ml^−1^ puromycin during 3 days, cells were treated with ionizing radiation (10 Gy) or mock-treated, incubated 1 h and then collected. Coverslips were washed with PBS, fixed on ice with methanol for 10 min followed by acetone for 30 s, washed three times with PBS and blocked with blocking solution from the Duolink PLA Kit for 30 min at 37 °C. Samples were incubated with primary antibodies against CCAR2 and CtIP (Supplementary Table 2) overnight at 4 °C, followed by MINUS and PLUS secondary PLA probes (antimouse minus and antirabbit plus) for 1 h at 37 °C. Detection was carried out with the Duolink Detection Kit Red (Olink Bioscience). At least 200 cells were analysed using a Leica Fluorescence microscope and foci counted automatically using command Granularity of the MetaMorph software.

### Immunoblotting

Extracts were prepared in Laemmli buffer (4% SDS, 20% glycerol and 125 mM Tris-HCl, pH 6.8), and proteins were resolved using SDS–PAGE and transferred to PVDF-LF membranes (Millipore), followed by immunoblotting. Western blot analysis was carried out using the antibodies listed in Supplementary Tables 2 and 3. Results were visualized using an Odyssey Infrared Imaging System (Li-Cor). Uncropped images of the most important blots are shown in Supplementary Figures.

### Single-molecule analysis of resection tracks

SMART was performed as described^18^. Briefly, U2OS cells downregulated for the indicated genes were grown in the presence of 10 μM BrdU for 24 h. Cultures were then irradiated (10 Gy) and harvested after 1 h. Cells were embedded in low-melting agarose (Bio-Rad), followed by DNA extraction. To stretch the DNA fibres, silanized coverslips (Genomic Vision) were dipped into the DNA solution for 15 min and pulled out at a constant speed (250 μm s^−1^). Coverslips were baked for 2 h at 65 °C and incubated directly without denaturation with an anti-BrdU mouse monoclonal antibody (Supplementary Table 2). After washing with PBS, coverslips were incubated with the secondary antibody (Supplementary Table 3). Finally, coverslips were mounted with ProLong Gold Antifade Reagent (Molecular Probes) and stored at −20 °C. DNA fibres were observed with a Nikon NI-E microscope and a PLAN FLOUR 40 × 0.75 PHL DLL objective. The images were recorded and processed with the NIS ELEMENTS Nikon Software. For each experiment, at least 200 DNA fibres were analysed, and the length of DNA fibres was measured with Adobe Photoshop CS4 Extended version 11.0 (Adobe Systems Incorporated).

### Protein expression and purification

Recombinant proteins were expressed in *Escherichia coli* BL21 (DE3). A fresh single transformant colony was inoculated into 5 ml of LB medium containing kanamycin (30 μg ml^−1^) for His_6_-CCAR2, or ampicillin (50 μg ml^−1^) for pGEX-CtIP constructs, and the cultures were incubated at 37 °C overnight with shaking. A 2.5 ml aliquot of the overnight culture was inoculated into 250 ml of LB medium containing the appropriate antibiotic and incubated at 23 °C (for His_6_-CCAR2) or 30 °C (for GST-CtIP constructs), until *A*_600_ reached 0.7. Expression was induced by adding isopropyl-1-thio-D-galactopyranoside (IPTG, Duchefa Biochimie). The final concentration of IPTG was 1 mM for CCAR2 and 0.1 mM for CtIP fragments. At 3 h after induction, cells were collected by centrifugation at 13,000*g* for 30 min, and the bacterial pellets were frozen immediately at −80 °C. For His_6_-CCAR2, the stored pellet was thawed and resuspended in sonication buffer (20 mM Tris-HCl, pH 8, 500 mM NaCl, 20% glycerol, 15 mM β-mercaptoethanol and 1% Tween-20). For CtIP fragment purification, the pellets were thawed and resuspended in PBS. Cells were disrupted by sonication, and the lysate was clarified by centrifugation. For His_6_-CCAR2 purification, supernatant was loaded onto a Ni^2+^-sepharose column (His-Trap HP columns, GE Healthcare) that had been pre-equilibrated with sonication buffer (SB). The column was washed with 10 ml of SB supplemented with 60 mM imidazole, eluted with a 30 ml gradient of imidazole (at 60 mM to 1 M) in SB and then collected in 0.5 ml fractions. For CtIP purification, the supernatant was loaded on a GSTrap HP column (GE Healthcare) pre-equilibrated with PBS. The columns were washed with PBS and eluted with 50 mM Tris-HCl, pH 8 and 10 mM reduced glutathione.

An aliquot of each fraction from the purifications was analysed by SDS–PAGE, and those containing the overexpressed protein were pooled and dialysed against either dialysis buffer 1 (50 mM Tris-HCl, pH 8.0, 500 mM NaCl, 1 mM dithiothreitol and 50% glycerol) for CCAR2, or dialysis buffer 2 (PBS, 30% glycerol) for CtIP and its deletion fragments. The protein preparation was divided into aliquots and stored at −80 °C. Protein concentrations were determined by the Bradford assay, and denatured proteins were analysed by SDS–PAGE.

### Pull-down assay using purified proteins

Eighty pmol of purified GST alone, GST-CtIP or GST fused to CtIP fragments were resuspended in a final volume of 300 μl with PBS, mixed with 100 μl of pre-equilibrated glutathione sepharose 4b resin (GE Healthcare) and incubated for 1 h at 4 °C. The resin was washed twice with binding buffer (20 mM Tris, pH 7.5, 1 mM EDTA, 10 mM beta-mercaptoethanol, 0.5% Triton and 50 mM NaCl). One hundred pmol of purified His_6_-CCAR2 was incubated at 4 °C for 1 h with either GST or GST-tagged proteins bound to resin. The matrix was washed twice with wash buffer (binding buffer with 3 mM reduced glutathione). Bound proteins were separated by SDS–PAGE (7.5%), transferred to polyvinylidene difluoride (PVDF) membranes and analysed by western blot analysis using antibodies against CCAR2.

### Pull-down assay from whole-cell extracts

Protein extracts from cells transfected with the different versions of GFP-CCAR2 were prepared in lysis buffer (20 mM Tris, pH 7.5, 1 mM EDTA, 0.5% Triton, 50 mM NaCl, 1 × protease inhibitors (Roche) and 1 × phosphatase inhibitor cocktail 1 (Sigma)). The amount of expression of each CCAR2 fragment was calculated by western blotting. Similar amounts of each CCAR2 truncated version were used for pull-down assays. After adding beta-mercaptoethanol (final concentration 10 mM) to the samples, cell extracts were pre-cleared by incubating with 50 μl of pre-equilibrated glutathione sepharose 4B resin (GE Healthcare) for 1 h at 4 °C.

Eighty pmol of purified GST-CtIP were resuspended in a final volume of 500 μl with PBS, mixed with 100 μl of pre-equilibrated glutathione sepharose 4B resin and incubated for 1 h at 4 °C. The resin was washed twice with binding buffer (lysis buffer with 10 mM beta-mercaptoethanol but without protease and phosphatase inhibitors) and then incubated with the pre-cleared cell extracts for 2 h at 4 °C. The matrix was washed twice with wash buffer (binding buffer with 3 mM reduced glutathione), and proteins were eluted by boiling the slurry for 5 min in protein-loading buffer. Precipitated proteins were separated by SDS–PAGE, transferred to PVDF membranes and analysed by western blot analysis.

### Immunoprecipitation

U2OS cells overexpressing GFP, GFP-CtIP or GFP-CCAR2 were irradiated (10 Gy) or mock-treated and harvested 1 h later in lysis buffer (50 mM Tris-HCl pH 7.5, 50 mM NaCl, 0.2% Triton X-100, 1 × protease inhibitors (Roche) and 1 × phosphatase inhibitor cocktail 1 (Sigma)). Protein extract (900 μg) was mixed with 30 μl of pre-equilibrated magnetic anti-GFP resin (GFP-Trap_M, Chromotek) and incubated overnight at 4 °C by gently rocking. Beads were then washed three times with lysis buffer, and the precipitate was eluted in 40 μl of Laemmli buffer.

### Chromatin immunoprecipitation

ChIP assays were carried out essentially as described^44^. Briefly, DiVA cells harbouring the nuclease AsiSI fused to the oestrogen receptor were incubated for 4 h with 300 nM of tamoxifen (4-OHT) to induce the translocation of the nuclease to the nucleus and the induction of DSBs. Cells expressing shRNA against CCAR2 or shRNA non-target were used as a control. Chromatin (300 μg) was immunoprecipitated with 2 μg of anti-RPA, anti-CtIP and anti-IgG (mock; Supplementary Table 2). The enrichment of specific DNA loci was analysed in immunoprecipitated chromatin and the input in triplicates by quantitative reverse transcriptase PCR (qRT–PCR). Primers are listed in Supplementary Table 4.

### Statistical analysis

Statistical significance was determined with a paired Student's *t*-test using the PRISM software (Graphpad Software Inc.). Statistically significant differences were labelled with one, two or three asterisks if *P*<0.05, *P*<0.01 or *P*<0.001, respectively. A Mann–Whitney test was used to detect statistically significant differences between the populations of resected DNA ends detected by SMART.

### Data availability

The authors declare that all data supporting the findings of this study are available within the article (and its Supplementary Information files) and upon request.

## Additional information

**How to cite this article:** López-Saavedra, A. *et al*. A genome-wide screening uncovers the role of CCAR2 as an antagonist of DNA end resection. *Nat. Commun.* 7:12364 doi: 10.1038/ncomms12364 (2016).

## Supplementary Material

Supplementary Information Supplementary Figures 1-5 and Supplementary Tables 1-4

Supplementary Data 1 Raw data obtained in the SSR screening. Genes are ordered alphabetically and those that have been selected as members of the sets that favor NHEJ or those that facilitates HR are marked in red and green respectively.

Supplementary Data 2 List of candidates that alter the NHEJ/HR balance.

Supplementary Data 3 List of candidates that alter the NHEJ/HR balance corrected by cell cycle distribution.
