# Supplementary material for: A genome-wide screening uncovers the role of CCAR2 as an antagonist of DNA end resection
Source: Nat Commun. 2016 Aug 9;7:12364. doi: 10.1038/ncomms12364 (PMC4980490; doi:10.1038/ncomms12364)
Supplement: Supplementary Information — Supplementary Figures 1-5 and Supplementary Tables 1-4 [file ncomms12364-s1.pdf]

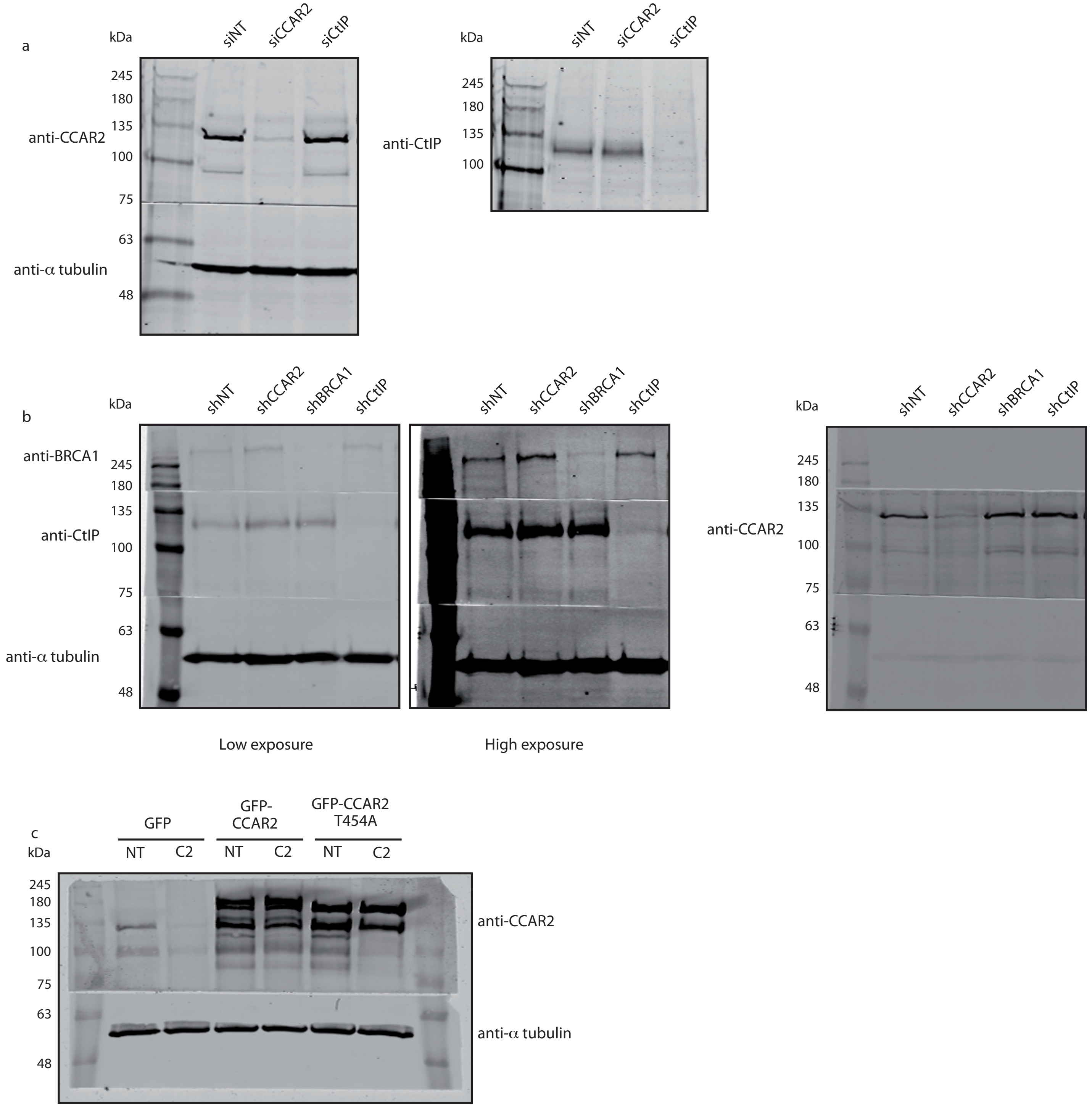

Supplementary Figure 1: Downregulation levels obtained with siRNA and shRNA of CCAR2, BRCA1 and CtIP. (a) Representative western blot showing the expression levels of CCAR2 and CtIP in cells transfected with the indicated siRNA. (b) Representative downregulation levels of CtIP, BRCA1 and CCAR2 obtained with shRNA. (c) Expression levels of GFP-CCAR2 and GFP-CCAR2-T454A in cells transfected with non-target (NT) siRNA or an siRNA against endogenous CCAR2.

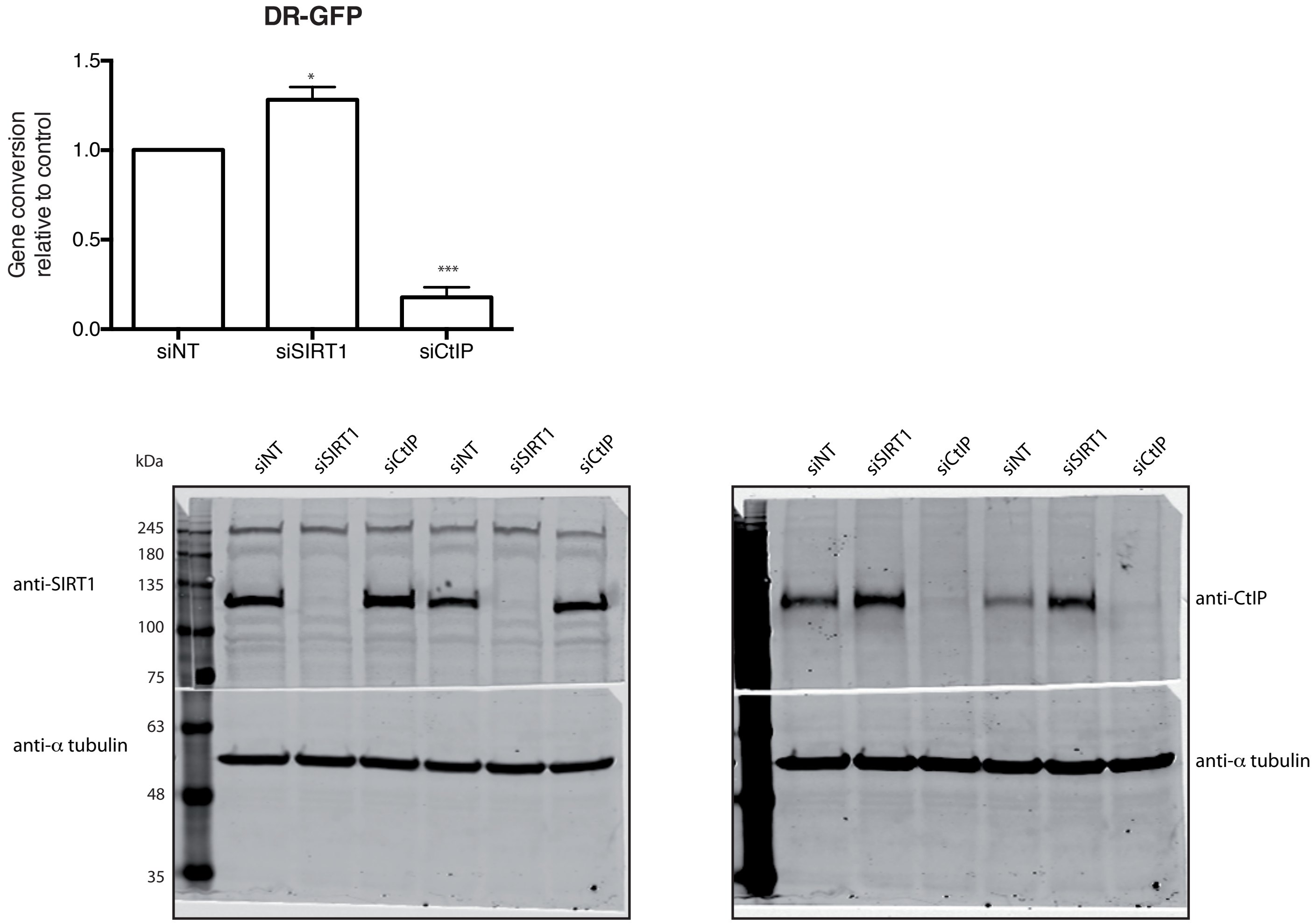

Supplementary Figure 2: Sirt1 contribution to homologous recombination. Effect of SIRT1 depletion on gene conversion, measured as GFP-positive cells in the DR-GFP reporter (left). The graph represent the average and standard deviation of three independent experiments. One representative western blot of two independent experiments showing the efficiency of SIRT1 downregulation with siRNA is shown.

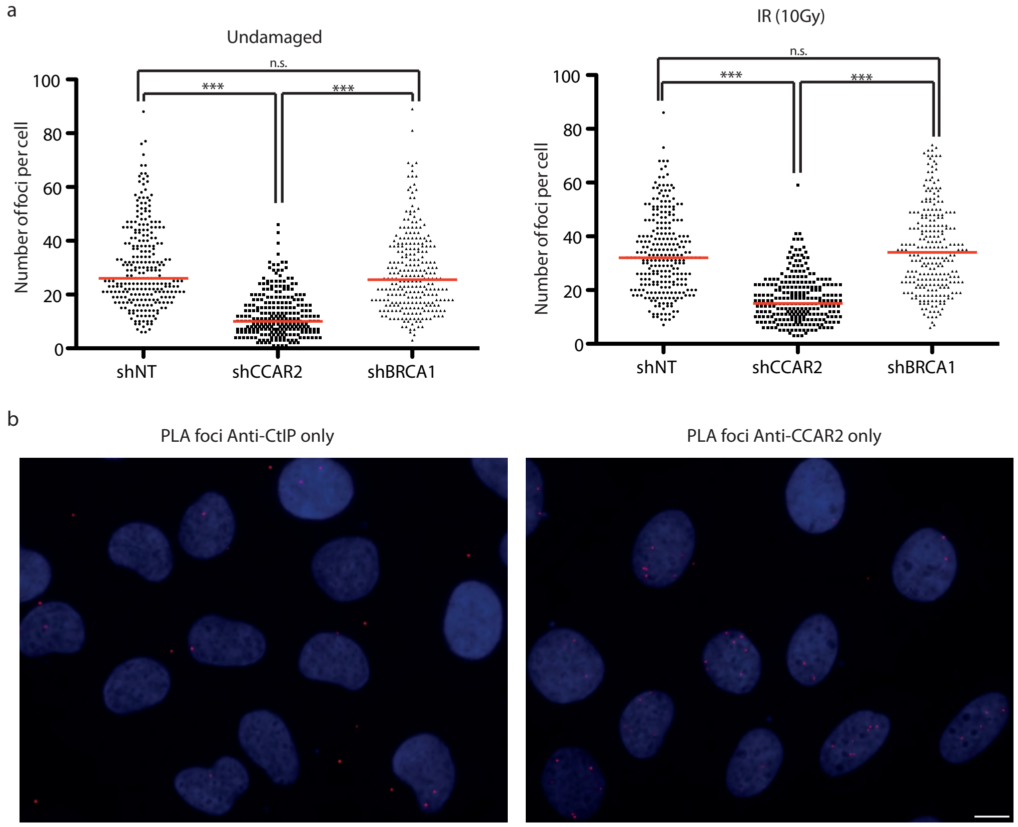

Supplementary Figure 3: PLA analysis of CtIP and CCAR2 interaction. (a) Individual results of cells in a representative experiment, with the median number of foci in red. The average of the medians and standard deviation of three experiments is shown in figure 5a. Left panel was obtained in cells untreated and right panel with cells exposed to 10Gy of ionizing radiation. (b) PLA signal obtained when only one antibody is used. The foci observed represent background levels of the experiment. White scale bar in the bottom left represent 7,5 μm.

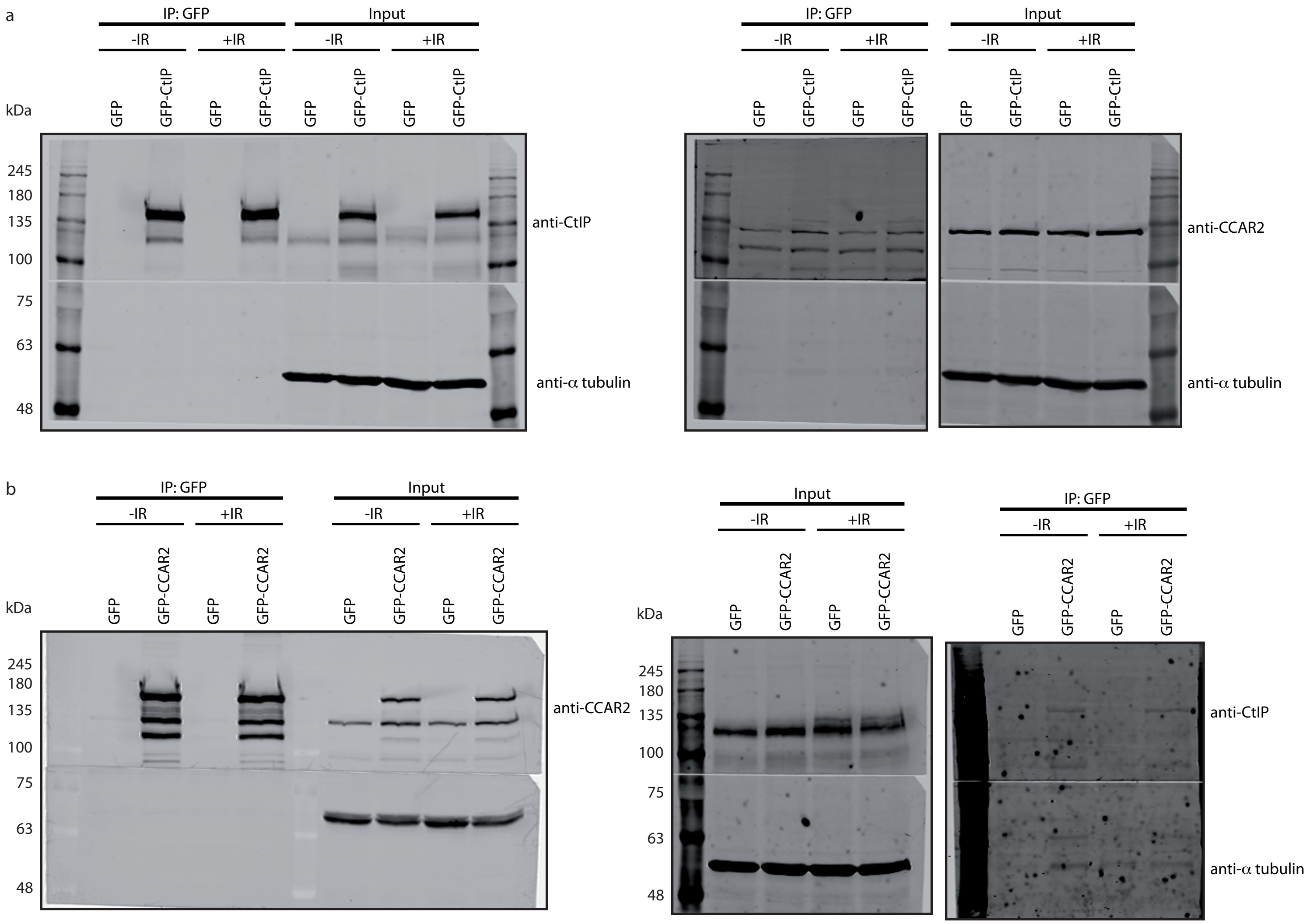

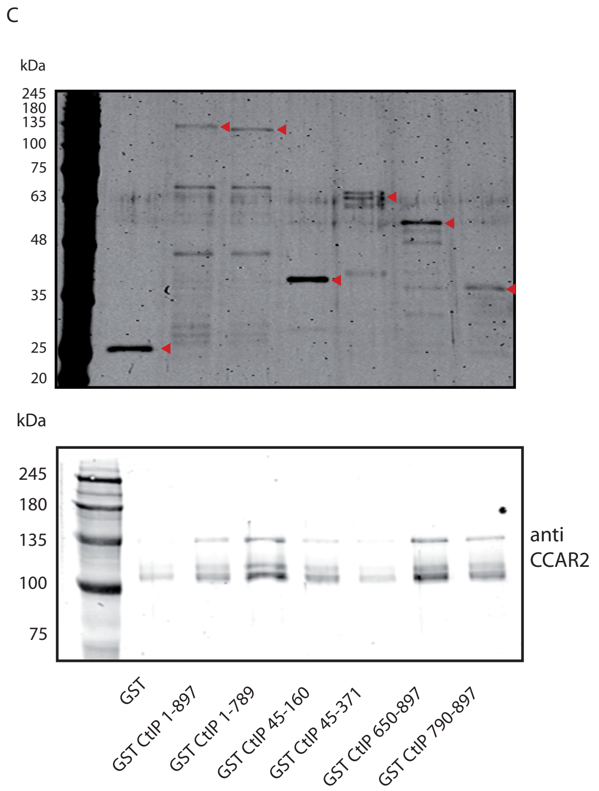

Supplementary Figure 4: Uncropped western blots of the data shown in Figure 5. (a) Uncropped blot of the immunoprecipitation shown in figure 5b b) Uncropped blot of the immunoprecipitation shown in figure 5b (c) Uncropped coomassie staining and western blot of the pull down shown in figure 5e

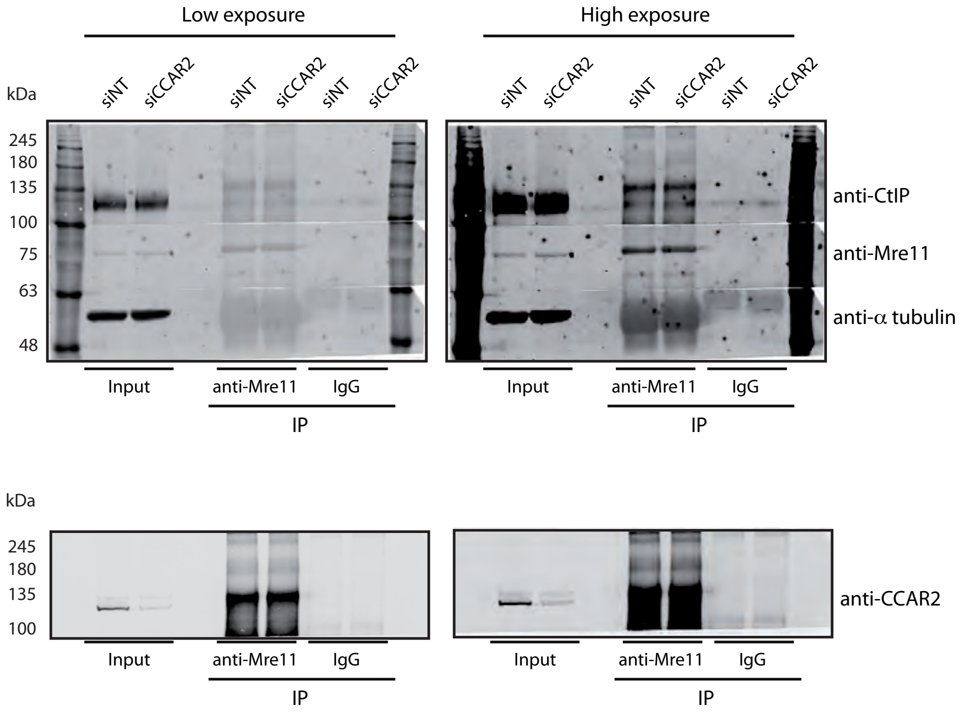

Supplementary Figure 5: CtIP-Mre11 interaction is not affected by CCAR2. Proteins were immunoprecipitated with an anti-Mre11 or IgG as control from U2OS cells expresing an shRNA against CCAR2 or a control shRNA and blotted with the indicated antibodies.

Supplementary Table 1: Mass spectrometry analysis of GFP-FLAG-CtIP interacting proteins. Protein samples from U2OS cells expressing GFP-3xFLAG-CtIP or control cells were purified using anti-FLAG resin. Upon elution with the 3xFLAG peptide, the samples were incubated with an anti-GFP matrix. Unbound complexes were washed and the proteins eluted by boiling in Laemmli buffer. Proteins were resolved by SDS-page, then silver stained and specific bands that appear in the GFP-3xFLAG-CtIP samples but not in the control were excised from the gel and sequenced by mass spectrometry. In total, we found 37 different peptides corresponding to CtIP (RBBP8) and 18 corresponding to CCAR2.

Accession	Mass	Score	Description
gi|41351350	103451	241	KIAA1967 [Homo sapiens]
gi|24432106	103465	234	protein KIAA1967 [Homo sapiens]
gi|193788217	103493	234	unnamed protein product [Homo sapiens]
gi|21739643	103324	224	hypothetical protein [Homo sapiens]
gi|18916825	92267	223	KIAA1967 protein [Homo sapiens]
gi|119584064	95317	222	KIAA1967, isoform CRA_c [Homo sapiens]
gi|1730321	103216	186	CtBP interacting protein CtIP [Homo sapiens]
gi|4506441	103190	186	DNA endonuclease RBBP8 isoform a [Homo sapiens]
gi|21040399	103906	186	RBBP8 protein [Homo sapiens]
gi|158258399	103105	178	unnamed protein product [Homo sapiens]
gi|42718017	99457	173	DNA endonuclease RBBP8 isoform b [Homo sapiens]
gi|119584063	67691	155	KIAA1967, isoform CRA_b [Homo sapiens]

Supplementary Table 2: Primary Antibodies used in this study. IF, immunofluorescence. WB, Western blotting. SMART, Single Molecule Analysis of Resection Tracks. LM, Laser Microirradiation

Target protein	Application	Supplier	Reference	Dilution
**RPA32**	IF	Abcam	ab2175	1:500
**γ-H2AX**	IF	Cell Signaling	2577L	1:500
**γ-H2AX**	LM	Abcam	ab22551	1:500
**Tubulin**	WB	SIGMA	T9026	1:50000
**BrdU**	SMART	Amersham	RPN202	1:1000
**BRCA1**	WB	Santa Cruz	sc-6954	1:1000
**CCAR2**	PLA	Bethyl Laboratories	IHC-00135	1:200

**CCAR2**	WB, LM, IF	Bethyl Laboratories	A300-433A-1	1:1500, 1:100, 1:150
**CtIP**	WB, PLA, LM	R. Baer	14.1	1:500, 1:200, 1:100
**GFP**	WB	Santa Cruz	sc-8334	1:1000
**SIRT1**	WB	Novus	NB110-57573	1:1000

Supplementary Table 3: Secondary antibodies used in this study. IF, immunofluorescence. WB, Western blotting. SMART, Single Molecule Analysis of Resection Tracks, LM, Laser Microirradiation

Antibody	Application	Supplier	Reference	Dilution
**Alexa Fluor 594 goat anti-mouse**	IF, SMART	Invitrogen	A11032	1:1000
**Alexa Fluor 488 goat anti-rabbit**	IF	Invitrogen	A11034	1:1000
**Alexa Fluor 568 goat anti-mouse**	LM	Invitrogen	A11031	1:700
**Alexa Fluor 647 goat anti-rabbit**	LM	Invitrogen	A21245	1:700
**IRDye 680RD goat anti-mouse IgG (H+L)**	WB	Li-Cor	926-68070	From 1:5000 to 1:25000 (depending on the dilution of primary antibody)
**IRDye 800CW goat anti-rabbit IgG (H+L)**	WB	Li-Cor	926-32211	From 1:5000 to 1:25000 (depending on the dilution of primary antibody)

Supplementary Table 4: Primers used for ChIP.

	Forward primer	Reverse primer
**DSB-3_800bp**	GGCACCTCAACAGGTAGCAT	GCCTCTCTTCGATGCTTTTG
**DSB-**	GGAAGGAGGGGCTACTAGGG	GAAAGCCCCATTCAGTTTGA

**3_80bp**		
**DSB-3_gene**	CAAAGATGACCACAGCCTCA	CACACCCCAAGAAAAGAGGA
**DSB-III_800bp**	GGGACAGCGCGTACTTTG	TCGCTAGGCCCAGCAGTT
**DSB-III_80bp**	CCGTCCGTTACGTAGAATGC	GGGCGGGGATTATGTAATTT
**DSB-V_800bp**	GTCAGTATGGCCCCAGAGTC	ACGGCTGATGGACTTAGACG
**DSB-V_80bp**	CCTAGCTGAGGTCGGTGCTA	GAAGAGTGAGGAGGGGGAGT
